# Referred molecular testing as a barrier to optimal treatment decision making in metastatic non‐small cell lung cancer: Experience at a tertiary academic institution in Canada

**DOI:** 10.1002/cam4.6886

**Published:** 2024-02-05

**Authors:** Grace K. Grafham, Kenneth J. Craddock, Weei‐Yuarn Huang, Alexander V. Louie, Liying Zhang, David M. Hwang, Ambica Parmar

**Affiliations:** ^1^ Temerty Faculty of Medicine University of Toronto Toronto Ontario Canada; ^2^ Department of Laboratory Medicine and Molecular Diagnostics Sunnybrook Health Sciences Centre Toronto Ontario Canada; ^3^ Department of Laboratory Medicine and Pathobiology University of Toronto Toronto Ontario Canada; ^4^ Department of Radiation Oncology Sunnybrook Health Sciences Centre Toronto Ontario Canada; ^5^ MacroStat Inc. Toronto Ontario Canada; ^6^ Division of Hematology and Medical Oncology, Department of Medicine Sunnybrook Health Sciences Centre Toronto Ontario Canada

**Keywords:** biomarkers, in‐house testing, molecular testing, non‐small cell lung cancer, targeted therapy, turnaround time

## Abstract

**Background:**

Molecular testing is critical to guiding treatment approaches in patients with metastatic non‐small cell lung cancer (mNSCLC), with testing delays adversely impacting the timeliness of treatment decisions. Here, we aimed to evaluate the time from initial mNSCLC diagnosis to treatment decision (TTD) following implementation of in‐house *EGFR*, *ALK*, and PD‐L1 testing at our institution.

**Methods:**

We conducted a retrospective chart review of 165 patients (send‐out testing, *n* = 92; in‐house testing, *n* = 73) with newly diagnosed mNSCLC treated at our institution. Data were compared during the send‐out (March 2017–May 2019) and in‐house (July 2019–March 2021) testing periods. We performed a detailed workflow analysis to provide insight on the pre‐analytic, analytic, and post‐analytic intervals that constituted the total TTD.

**Results:**

TTD was significantly shorter with in‐house testing (10 days vs. 18 days, *p* < 0.0001), driven largely by decreased internal handling and specimen transit times (2 days vs. 3 days, *p* < 0.0001) and laboratory turnaround times (TAT, 3 days vs. 8 days, *p* < 0.0001), with 96% of in‐house cases meeting the international guideline of a ≤ 10‐day intra‐laboratory TAT (vs. 74% send‐out, *p* < 0.001). Eighty‐eight percent of patients with in‐house testing had results available at their first oncology consultation (vs. 52% send‐out, *p* < 0.0001), and all patients with in‐house testing had results available at the time of treatment decision (vs. 86% send‐out, *p* = 0.57).

**Conclusion:**

Our results demonstrate the advantages of in‐house biomarker testing for mNSCLC at a tertiary oncology center. Incorporation of in‐house testing may reduce barriers to offering personalized medicine by improving the time to optimal systemic therapy decision.

## Graphical abstract

Timely biomarker testing enables efficient initiation of optimal targeted treatments in metastatic non‐small cell lung cancer (mNSCLC) patients. We retrospectively compared time to treatment decision between mNSCLC patients with biomarkers tested in‐house (*n* = 73) and those sent‐out to an external laboratory (*n* = 92). We found that in‐house testing significantly decreased treatment decision times, resulting in a reduction in missed/suboptimal treatment opportunities.

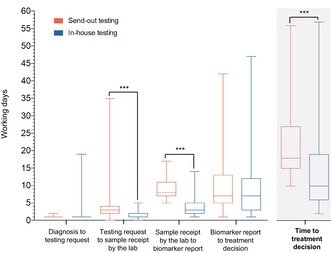

## 1 INTRODUCTION

Treatment paradigms for non‐small cell lung cancer (NSCLC) are increasingly driven by precision‐medicine approaches. For all stages of NSCLC, actionable mutation status influences decisions regarding adjuvant or palliative‐intent systemic therapy. For instance, identification of epidermal growth factor receptor (*EGFR*) positivity among patients with surgically resectable disease confers a benefit to targeted treatment with osimertinib in the adjuvant setting.^1^ In the metastatic setting, targeted therapies directed at EGFR and anaplastic lymphoma kinase (*ALK*) have consistently shown improved clinical outcomes compared to conventional chemotherapy.^2^, ^3^, ^4^, ^5^ For patients lacking actionable mutations, assessment of programmed death ligand‐1 (PD‐L1) expression levels are necessary to guide the effective use of immunotherapy drugs.^6^, ^7^, ^8^ As such, current international guidelines have recommended upfront routine molecular testing at the time of initial diagnosis as standard‐of‐care practice for all patients diagnosed with NSCLC.^9^

The College of American Pathologists/International Association for the Study of Lung Cancer/Association for Molecular Pathology guidelines on molecular testing for lung cancer recommend a turnaround time (TAT) of ≤10 working days from receipt of the specimen in the testing laboratory to release of the biomarker testing report.^10^ In a 2020 global survey, 29% of participating physicians reported delays of >10 days in receiving lung cancer biomarker results, with the highest percentages in the United States and Canada.^11^ Currently there is no established national approach to lung cancer biomarker testing in Canada leading to jurisdictional variation. With prior testing rates as low as 10% in certain regions, protracted TATs may serve as a major barrier in the decision‐making process for requesting biomarker tests, as well as in guiding effective systemic therapy decisions.^12^, ^13^

Multiple barriers to timely molecular testing have been previously identified, such as difficulty obtaining adequate tissue for analysis, inefficiencies in the ordering and reporting of results, and limitations in public funding to support routine comprehensive gene testing.^14^, ^15^, ^16^ In metastatic NSCLC (mNSCLC), increased time to testing is associated with delayed treatment initiation, lower application of appropriate systemic therapies, and increased mortality.^17^, ^18^ Implementation of reflex testing, whereby biomarkers are automatically requested by the pathologist at the time of initial tissue diagnosis, is one strategy shown to accelerate both TAT and time to optimal first‐line systemic therapy in mNSCLC.^19^, ^20^ This strategy was adopted at our institution in 2013 and is now considered the standard‐of‐care for patients with NSCLC.^9^, ^21^ A better understanding of the factors that contribute to testing delays would enable institutions to define and implement additional strategies to further improve biomarker testing times.

With this objective in mind, our institution approved and implemented in‐house *EGFR*, *ALK*, and PD‐L1 testing for NSCLC in July 2019. Previously, samples were sent‐out to a centralized reference laboratory for biomarker analysis. The use of in‐house laboratories is one method that has been demonstrated to shorten testing times; however, its impact on the remainder of the lung cancer diagnostic pathway is poorly understood.^19^, ^22^ In this study, we performed a detailed workflow analysis to determine whether in‐house biomarker testing improved the timeliness of treatment decisions for patients with mNSCLC.

## 2 METHODS

### 2.1 Patient selection

**FIGURE 1:**
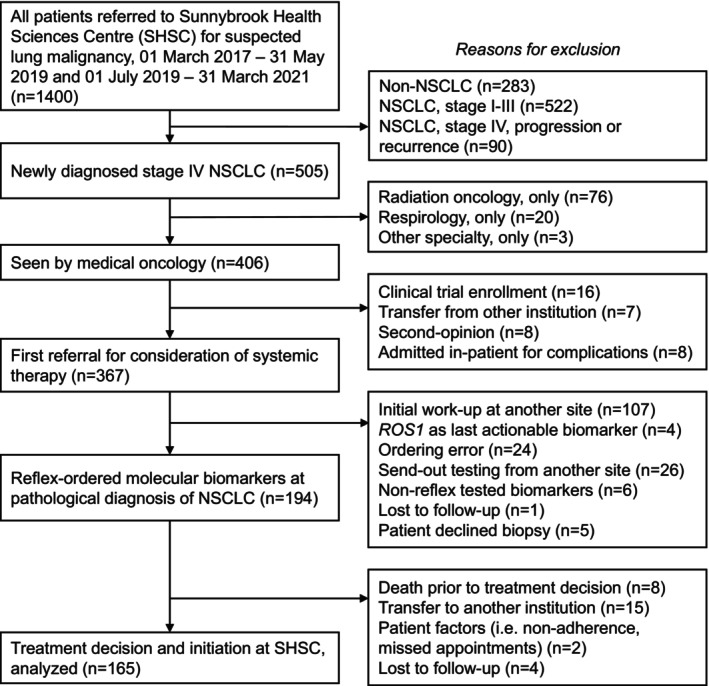
Flow diagram of patients analyzed.

We retrospectively reviewed patients with mNSCLC treated at Sunnybrook Health Sciences Centre (Toronto, Canada) between March 1, 2017 and March 31, 2021. Eligible patients were those aged ≥18 years with biopsy‐proven mNSCLC at initial assessment and reflex‐ordered biomarkers at the time of pathological diagnosis. Patients were excluded if their biopsy specimen and tissue diagnosis were obtained externally or if they were not assessed by a medical oncologist at our institution (Figure 1). In‐house *EGFR*, *ALK* and PD‐L1 testing was implemented at our institution on June 19, 2019. Prior to this, tissue samples were sent‐out to a centralized reference laboratory for biomarker assessment, consisting of *EGFR* qPCR and immunohistochemistry for *ALK* and PD‐L1. Included patients were divided into those with send‐out biomarker testing (March 1, 2017 to May 31, 2019) and in‐house testing at our institution (July 1, 2019 to March 31, 2021).

This study was performed in accordance with the Declaration of Helsinki and approved by the Sunnybrook Health Sciences Centre Research Ethics Board (SUN‐3774).

### 2.2 *EGFR* , *ALK* , and PD‐L1 testing

We investigated *EGFR*, *ALK*, and PD‐L1 single‐gene testing. These three targets were the standard‐of‐care, publicly funded biomarker panel in our jurisdiction during the study period. Patients whose treatment decisions were dependent on initial testing of exceptional biomarkers were excluded (Figure 1).

At the centralized reference laboratory, *EGFR* mutation testing was predominantly performed using the EGFR Mutation Analysis Kit (Entrogen, EGFR‐RT52). *ALK* immunohistochemistry was performed using the 5A4 antibody. Assessment of PD‐L1 tumor proportion scores (TPS) were completed using the 22C3 PharmDX Assay (Agilent).

At our institution, *EGFR* testing was performed using either the Idylla™ EGFR Mutation Test (Biocartis) or EGFR Mutation Analysis Kit (EGFR‐RT52, Entrogen). *ALK* immunohistochemistry was completed using the 1A4 antibody (Origene Technologies). PD‐L1 expression levels were determined using the 22C3 antibody on a Dako Omnis stainer.

### 2.3 Data collection and outcomes

Patient demographics, tumor pathology, and detailed timeline information were collected from patients' electronic medical records and our pathology laboratory information system. For each patient, the following dates were extracted: (1) pathological diagnosis of NSCLC, (2) pathologist request for biomarker testing, (3) sample receipt/accessioning at the send‐out or in‐house laboratory, (4) reporting of results by the send‐out or in‐house laboratory, (5) initial medical oncology consultation, and (6) treatment decision for systemic therapy.

The primary outcome was time to treatment decision (TTD) defined as the number of working days between the date of pathological diagnosis of mNSCLC and systemic therapy decision. We also examined the pre‐analytic, analytic, and post‐analytic intervals that constituted the total TTD. Patients whose treatment decisions were made prior to completed biomarker testing were not included in the workflow analysis. Additional outcomes included sample adequacy, biomarker results (i.e., positive, negative, and indeterminate), and the proportion of patients with results available at the initial oncology consultation and time of treatment decision.

### 2.4 Statistical analysis

All data were compared between the send‐out and in‐house testing cohorts. Categorical data were summarized by counts and proportions and compared using Fisher's exact test. Continuous data were described using median and interquartile range (IQR) and compared using Wilcoxon rank‐sum test. A two‐sided *p*‐value <0.05 was considered statistically significant. Statistical analyses were conducted using Statistical Analysis Software (SAS version 9.4, Cary, NC).

## 3 RESULTS

### 3.1 Patient and tumor characteristics

A total of 165 patients with newly diagnosed mNSCLC were included, 92 in the send‐out testing cohort and 73 in the in‐house testing cohort (Figure 1). Patient demographics were not statistically significant between cohorts (Table 1). The median age of all patients was 72 years (range, 40–95 years) and 48% (*n* = 80) were male. Most patients had lung adenocarcinoma (86%, *n* = 142), while 7% (*n* = 12) had squamous cell carcinoma, 0.6% (*n* = 1) had large cell carcinoma, and 6% (*n* = 10) had other/not otherwise specified pathology (Table 2). Molecular testing was most often performed on tissue biopsy or resection specimens (67%, *n* = 111). There was a higher proportion of patients with testing on cytology specimens in the send‐out cohort (42%, *n* = 39) compared to the in‐house testing cohort (21%, *n* = 53; *p* = 0.004).

**TABLE 1:** Baseline demographics and characteristics of metastatic NSCLC patients.

	Send‐out testing (*n* = 92)	In‐house testing (*n* = 73)	*p*‐value
Age at diagnosis, years, median (IQR)	73 (66, 81)	70 (62, 78)	0.23
Sex, male	46 (50)	34 (46.6)	0.75
Ethnicity			0.58
Caucasian	40 (43.5)	25 (34.2)	
Asian	23 (25)	24 (32.9)	
Black	3 (3.3)	1 (1.4)	
Other	4 (4.3)	2 (2.7)	
Unknown	22 (23.9)	21 (28.8)	
Smoking status			0.71
Current	20 (21.7)	19 (26)	
Former	42 (45.7)	34 (46.6)	
Never	30 (32.6)	20 (27.4)	
ECOG performance status^a^			0.11
0–1	48 (52.2)	44 (60.3)	
≥2	37 (40.2)	19 (26)	
Unknown	7 (7.6)	10 (13.7)	
Age‐adjusted CCI			0.21
≤3	44 (47.8)	27 (37)	
>3	48 (52.2)	46 (63)	

Abbreviations: CCI, Charlson Comorbidity Index; ECOG, Eastern Cooperative Oncology Group; IQR, interquartile range.

^a^ ECOG performance status was recorded at the time closest to treatment decision. Data are presented as *n* (%) unless indicated otherwise. Statistical significance, two‐sided *p* < 0.05.

**TABLE 2:** Pathological tumor and molecular biomarker testing characteristics.

	Send‐out testing (*n* = 92)	In‐house testing (*n* = 73)	*p*‐value
Histology			0.63
Adenocarcinoma	79 (85.9)	63 (86.3)	
Squamous cell carcinoma	8 (8.7)	4 (5.5)	
Large cell carcinoma	0 (0)	1 (1.4)	
Other/NOS	5 (5.4)	5 (6.8)	
Specimen type			0.004
Cytology	39 (42.4)	15 (20.6)	
Tissue biopsy/resection	53 (57.6)	58 (79.4)	
Sample inadequate for testing	11 (11.9)	5 (6.9)	0.27
*EGFR*			0.69
Positive	32 (34.8)	25 (34.2)	
Negative	50 (54.3)	44 (60.3)	
Indeterminate	3 (3.3)	1 (1.4)	
Not ordered	7 (7.6)	3 (4.1)	
*ALK*			0.34
Positive	6 (6.5)	2 (2.7)	
Negative	79 (85.9)	67 (91.8)	
Indeterminate	0 (0)	1 (1.4)	
Not ordered	7 (7.6)	3 (4.1)	
PD‐L1			0.013
<1%	32 (34.8)	20 (27.4)	
1%–49%	13 (14.1)	19 (26)	
≥50%	36 (39.1)	33 (45.2)	
Not ordered	11 (12)	1 (1.4)	

*Note*: Data are presented as *n* (%). Statistical significance, two‐sided *p* < 0.05. EGFR and *ALK* were not routinely tested in squamous cell carcinoma.

Abbreviations: *ALK*, anaplastic lymphoma kinase; *EGFR*, epidermal growth factor receptor; NOS, not otherwise specified; PD‐L1, programmed death‐ligand 1.

### 3.2 Biomarker testing patterns

The frequency of *EGFR* mutations detected in patients tested was around 34% in both cohorts (send‐out testing, *n* = 32; in‐house testing, *n* = 25), and for *ALK*‐positive cases was 7% (*n* = 6) in the send‐out testing cohort and 3% (*n* = 2) in the in‐house testing cohort (Table 2). The send‐out cohort had a higher proportion of cases with PD‐L1 TPS <1% compared with in‐house testing (35% vs. 27%), and lower proportions of cases with TPS 1–49% (14% vs. 26%) and ≥ 50% (39% vs. 45%, *p* = 0.013). Patients with send‐out testing also had 11%‐point fewer PD‐L1 tests ordered overall (*p* = 0.013), as validation of PD‐L1 testing on cytology cell block preparations was not completed until near the end of the send‐out testing period.

### 3.3 Sample quality

Eleven patients (12%) in the send‐out cohort and five patients (7%) in the in‐house cohort had unsuccessful biomarker testing due to insufficient tissue, suboptimal specimen quality, and/or sample processing issues (*p* = 0.27; Table 2). Of those with inadequate samples, one patient in the send‐out cohort and two patients in the in‐house cohort were too ill for repeat tissue biopsy and were alternatively eligible for enrolment in a clinical trial that offered plasma‐based molecular testing.^23^ All remaining patients in either cohort underwent a second procedure to acquire additional tissue for analysis. This second procedure was successful for seven out of ten send‐out testing patients and three out of three in‐house testing patients. Two‐third of the remaining send‐out patients underwent a third procedure of which was successful in acquiring additional tissue. For these three patients, one proceeded with standard chemotherapy and two proceeded with best supportive care (BSC) consisting of appropriate palliative care without any anticancer therapies.

### 3.4 Availability of biomarker results

**FIGURE 2:**
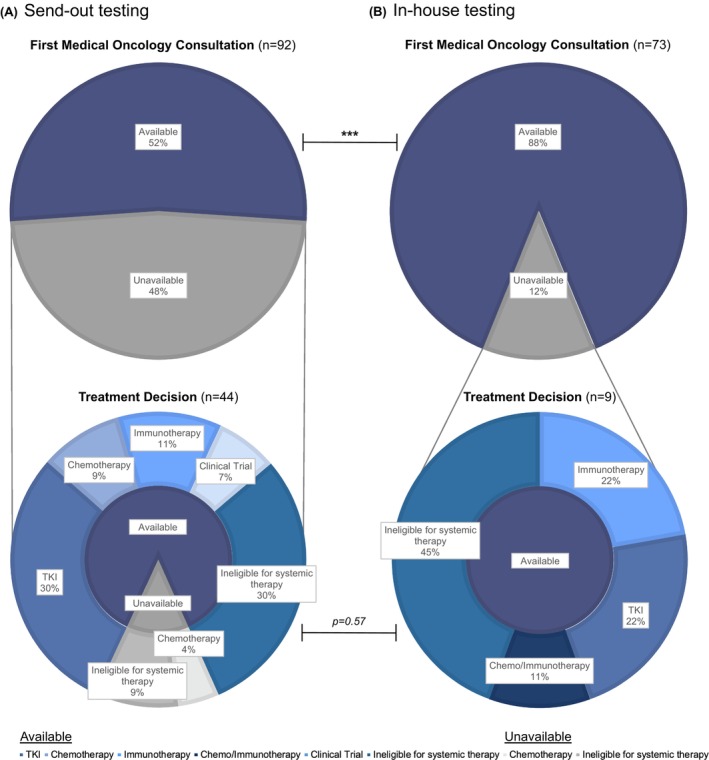
Distribution of molecular biomarker testing results and treatment decisions for the (A) send‐out and (B) in‐house testing cohorts. The upper pie charts depict the availability of results at patients' first medical oncology consultation, while the lower pie charts depict the availability of results at the time of final treatment decision for patients whose results were unavailable in the former. The total percentage of available versus unavailable biomarker results were compared between the send‐out and in‐house testing cohorts. Statistical significance, p < 0.0001***. TKI, tyrosine kinase inhibitor.

Of patients with in‐house biomarker testing, 88% (*n* = 64) had test results for their last actionable biomarker available at their initial medical oncology consultation compared to only 52% (*n* = 48) of patients with send‐out testing (*p* < 0.0001; Figure 2). The last actionable biomarker denotes the final biomarker outcome necessary to make an informed treatment decision regarding first‐line systemic treatment (i.e., tyrosine kinase inhibitors for *EGFR* and *ALK* mutations, and immunotherapy with or without chemotherapy as dictated by PD‐L1 expression levels). All remaining patients with in‐house testing had results for their last actionable biomarker available at the time of treatment decision (*vs*. 86%, *n* = 38 with send‐out testing; *p* = 0.57). Six patients with send‐out testing had treatment decisions made after tissue samples were sent for biomarker testing but prior to receiving results for their last actionable biomarker. Two patients initiated treatment with chemotherapy alone, while the remaining four clinically deteriorated while awaiting results and were consequently ineligible for systemic therapy opting to pursue best supportive care alone. Biomarker testing was ultimately found to be negative for any actionable mutation for the two patients who initiated treatment with chemotherapy prior to receipt of results.

### 3.5 Workflow analysis

**FIGURE 3:**
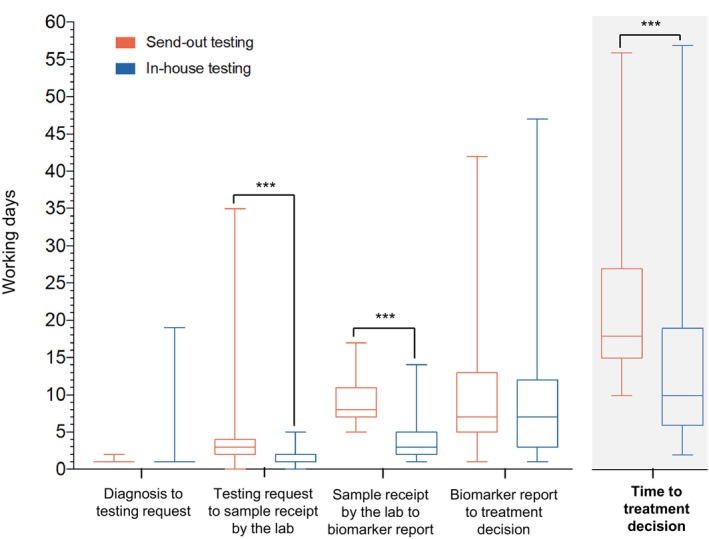
Time to treatment decision workflow analysis. Box and whisker plots comparing time to treatment decision (TTD) and the individual intervals constituting the total TTD between the send‐out and in‐house testing cohorts. Each box spans the interquartile range (IQR) with the median depicted by the horizontal central line. Whiskers extend to the minimum and maximum values. Time intervals are measured in number of working days. Patients whose treatment decisions were made prior to completion of the final biomarker report were excluded. No box is noted for diagnosis to testing request as median and IQR is 1 working day for both cohorts, given the reflex testing policy implemented at our institution. Summary data are shown in Table 3. Statistical significance, p < 0.0001***.

There was a significant improvement in TTD with the implementation of in‐house biomarker testing from a median of 18 (IQR, 15–27 days) to 10 working days (IQR, 6–19 days; *p* < 0.0001). To provide insight on the time‐points that constituted the total TTD, we performed a detailed workflow analysis of the pre‐analytic, analytic, and post‐analytic intervals (Table 3, Figure 3).

**TABLE 3:** Timeliness of treatment decisions for metastatic NSCLC patients.

	Send‐out testing (*n* = 86)	In‐house testing (*n* = 73)	*p*‐value
Time to treatment decision, working days, median (IQR)	18 (15, 27)	10 (6, 19)	<0.0001
Diagnosis to testing request	1 (1, 1)	1 (1, 1)	0.85
Testing request to sample receipt by the lab	3 (2, 4)	2 (1, 2)	<0.0001
Sample receipt by the lab to biomarker report	8 (7, 11)	3 (2, 5)	<0.0001
≤10 days, *n* (%)	64 (74.4%)	70 (95.9%)	0.0002
>10 days, *n* (%)	22 (25.6%)	3 (4.1%)	
Biomarker report to treatment decision	7 (5, 13)	7 (3, 12)	0.82

*Note*: Patients with treatment decisions made prior to release of their final biomarker report were excluded. Data are presented as median (IQR) unless indicated otherwise. Statistical significance, two‐sided *p* < 0.05.

Abbreviation: IQR, interquartile range.

The pre‐analytic interval included the time from pathological diagnosis to biomarker testing request, and specimen transit time. The median time between sign‐out of the diagnostic pathology report and opening of the biomarker case file in our institution's laboratory information system was 1 working day (IQR, 1 day) for both cohorts. The exceptional case in the in‐house cohort, requiring 19 working days, pertains to a case in which the tissue diagnosis had been established but the pathologist refrained from ordering biomarkers due to suboptimal specimen quality. Median specimen transit time, from biomarker testing request to the receipt and accessioning of the specimen in the laboratory for analysis, was faster for in‐house versus send‐out testing (2 days [IQR, 1–2 days] vs. 3 days [IQR, 2–4 days]; *p* < 0.0001).

The analytic interval or laboratory TAT is the time between receipt of the sample in the laboratory for biomarker analysis and release of the final biomarker report by the pathologist. With in‐house testing, median laboratory TAT significantly decreased from 8 (IQR, 7–11 days) to 3 working days (IQR, 2–5 days; *p* < 0.0001), with 96% of cases meeting the current CAP/IASLC/AMP guidelines of a ≤10 working day TAT versus 74% of cases with send‐out testing (*p* = 0.0002).

In‐house testing did not impact the post‐analytic interval, from the release of the final biomarker report to the treatment decision by the medical oncology team (Table 3).

## 4 DISCUSSION

In the present study, we found that implementation of in‐house biomarker testing for mNSCLC led to a significant reduction in TTD compared to send‐out testing. This was largely driven by improvements in the pre‐analytic and analytic testing intervals. Additionally, there was an increase in the number of patients with biomarker test results available at their initial medical oncology assessment. This resulted in a complete reduction in missed treatment opportunities, where no patients with in‐house testing started treatment with conventional chemotherapy alone before biomarker results were available. Together, our findings highlight the potential diagnostic, clinical and therapeutic utility of in‐house *EGFR*, *ALK*, and PD‐L1 testing for mNSCLC.

The role of molecular testing in oncology care is rapidly evolving. Molecular testing is required to identify actionable mutations that are being identified in a growing proportion of NSCLC patients, with assessment of PD‐L1 expression levels guiding the use of immunotherapy agents in the remaining patient population.^7^, ^24^, ^25^ Time to treatment decision reflects the multidisciplinary approach to lung cancer—from the detection of sensitizing mutations by the pathology laboratory to the identification of optimal treatment choices by the medical oncologist. However, factors contributing to delays in treatment decisions have been inadequately characterized, particularly in cases where biomarker testing is performed by laboratories external to where the patient is being treated. Our study aimed to address this knowledge gap by demonstrating improved treatment decision times with in‐house biomarker testing, with specific consideration of the component intervals contributing to the total TTD.

The CAP/IASLC/AMP guidelines suggest that patients with advanced NSCLC have completed testing for actionable driver alterations within 10 working days of specimen receipt in the testing laboratory.^26^ A 2015 Q‐probes survey from CAP found that 78% of the 26 participating institutions met the 10‐day target (median, 8 days), with majority of *EGFR* and *ALK* tests being sent‐out.^27^ Similarly, DiStasio et al reported that 82% of send‐out cases from their large academic center were available within the target interval (median, 9 days).^28^ Our results show that in‐house biomarker testing has the potential for even faster TATs (median, 3 days with 96% of cases ≤10 day TAT). Our shorter in‐house TATs may be attributed in part to faster testing methods, specifically our in‐house qPCR assay which no longer required 2 days of DNA extraction prior to testing.^29^ Nonetheless, even if the same assays were used, our final hypothetical in‐house TAT of 5 days would still be shorter than the 8 day send‐out TAT. This suggests that TAT is influenced by factors beyond testing method which may include lower backlogs of specimens requiring analysis with in‐house as compared to send‐out, centralized testing. Accordingly, decisions regarding testing methods, optimal testing center numbers, and locations should take into consideration expected volumes of NSCLC patients and testing capacity in the region.

The decrease in specimen transit time was an expected benefit of testing in‐house. In contrast to laboratory TAT which typically shows low variability, the transportation phase for send‐out testing is highly variable.^28^ For our send‐out cohort, specimen transit was the most variable interval despite our institution using a direct courier service. In the literature, nonanalytic delays are consistently cited as the primary cause of lagging treatment times with as many as 87% of pathology‐related adverse events being due to pre‐analytic error.^16^, ^30^, ^31^ This broadly encompasses interruptions in test ordering and specimen collection, transport, accessioning, and processing. In both testing settings, factors like staffing distribution and workflow must be considered. Like others, we observed extended pre‐analytic times for in‐house biomarker requests made towards the end of the workweek, a likely consequence of high specimen volumes at the onset of the following workweek as our laboratory only accessions specimens for biomarker testing on weekdays.^28^ Nonetheless, the growing complexity of biomarker testing with added handoffs to specialized laboratories increases the risk of pre‐analytic delays, especially for institutions that are physically distant from the nearest testing laboratory.^16^ These modifiable factors that contribute to delays in treatment decision require attention to ensure improvements in TAT are not offset by nonanalytic factors.^19^

Delays in biomarker testing results are a challenge to personalized medicine given the risk of clinical deterioration of the patient.^32^ For this reason, some oncologists may consider treatment with conventional chemotherapy alone before biomarker results are available to ensure the window‐of‐opportunity to initiate treatment is not lost. A previous review from our institution in the send‐out testing era, found that mNSCLC patients had faster treatment times if biomarker test results were available at their initial oncology assessment, although this represented only 27% of patients in that study.^20^ In contrast, among our cohort, 88% of patients with in‐house testing had results available at their first oncology consultation and all patients had results available at the time of treatment decision. This subsequently led to faster treatment decisions and a complete reduction in missed opportunities for first‐line targeted treatment or immunotherapy. This emphasizes the critical importance of continuing to reduce testing wait times to ensure timely treatment initiation in mNSCLC patients.

A major barrier to in‐house testing is financial cost. Material costs alone for a single‐gene test range from $133–200 Canadian dollars (CAD) per sample and can increase up to $652 CAD when operational costs are included.^33^ As the breadth of clinically relevant biomarkers for mNSCLC continues to expand, testing strategies have shifted from single‐gene testing to multiplex comprehensive testing, namely by next‐generation sequencing (NGS). A recent economic model among Canadian patients with mNSCLC showed that molecular testing with NGS had the lowest total cost of testing per patient relative to all combined single‐gene testing strategies across identical biomarkers ($3480 vs. $5632 CAD, excluding operational costs), with delayed care having the largest influence on total cost.^34^ While the average TAT for comprehensive NGS reporting is greater than 2 weeks, augmented in‐house NGS strategies have demonstrated TATs comparable to those observed in the current study—2 to 3 working days using an integrated ultrafast NGS approach.^35^, ^36^, ^37^ Nonetheless, access to in‐house NGS remains restricted to subspecialized facilities with many jurisdictions unable to afford the infrastructure costs associated with routine reflex NGS. For oncology patients with limited diagnostic samples and those in jurisdictions with restricted funding, single‐gene testing remains clinically relevant for maximizing availability of biomarker results to help guide treatment decisions, within the constraints of available specimens and/or resources.

A limitation of our retrospective study is the evaluation of only three single‐gene assays (*EGFR*, *ALK*, and PD‐L1) during the study period, in comparison to the expanded range of actionable genomic alterations currently evaluated as standard‐of‐care practice in non‐squamous NSCLC. These three targets were evaluated as the standard‐of‐care, publicly funded biomarker panel in our jurisdiction during the study period, apart from ROS1 which was funded towards the end of the study period and thus, excluded from our analysis to maintain consistency between cohorts.^26^ Our jurisdictional policies have since upgraded to meet the international standard for clinical care, comprising of in‐house NGS testing at initial non‐squamous NSCLC diagnosis for *EGFR*, *ALK*, *ROS‐1*, as well as v‐raf murine sarcoma viral oncogene homolog B (*BRAF*), erb‐B2 receptor tyrosine kinase 2 (*ERBB2*), kirsten ras oncogene homolog (*KRAS*), phosphatidylinositol 4,5‐bisphosphate 3‐kinase catalytic subunit alpha isoform (*PIK3CA*), fibroblast growth factor receptor 1 (*FGFR1*), met proto‐oncogene, receptor tyrosine kinase (*MET*), neurotrophic tyrosine receptor kinase 1–3 (NTRK1‐3), ret proto‐oncogene (*RET*), and PD‐L1 expression by IHC.^26^, ^38^, ^39^ Despite this, we identified challenges that may also be faced in the current era of NGS testing through the use of a small number of centralized laboratories: poor pre‐analytic quality assurance, lengthy TATs, and disjointed communication between clinicians.^14^, ^40^, ^41^

Our results highlight the advantages of in‐house biomarker testing for mNSCLC at a tertiary oncology center. Although further studies are necessary to evaluate its economic and survival impact, in‐house testing has shown the potential to reduce barriers to personalized medicine by improving biomarker testing efficiency and the time to optimal systemic therapy decision.

## AUTHOR CONTRIBUTIONS

**Grace K. Grafham:** Conceptualization (equal); data curation (equal); investigation (equal); methodology (equal); visualization (equal); writing – original draft (equal). **Kenneth J. Craddock:** Writing – review and editing (equal). **Weei‐Yuarn Huang:** Writing – review and editing (equal). **Alexander v. Louie:** Writing – review and editing (equal). **Liying Zhang:** Formal analysis (equal); visualization (equal); writing – review and editing (equal). **David M. Hwang:** Conceptualization (equal); methodology (equal); supervision (equal); writing – review and editing (equal). **Ambica Parmar:** Conceptualization (equal); methodology (equal); project administration (equal); supervision (equal); writing – review and editing (equal).

## FUNDING INFORMATION

This work was supported by the Anatomic Pathology Partnership of Sunnybrook (APPS) academic fund. The funders had no role in the study design, data collection and analysis, decision to publish, or preparation of the manuscript.

## CONFLICT OF INTEREST STATEMENT

WH reports receiving honoraria as a speaker for Amgen Canada Inc. and Bayer AG; participating in advisory boards for Bayer AG and Pfizer Inc.; and grant funding from Pfizer Inc. AVL reports receiving honoraria as a speaker and participating on the advisory board for AstraZeneca Canada Inc. DMH reports receiving honoraria as a speaker for Merck & Co Inc., F Hoffman‐La Roche AG, Eli Lilly and Company, Novartis AG, AstraZeneca Canada Inc., Pfizer Inc., and GSK; participating in advisory boards for Novartis AG, Merck & Co Inc., Amgen Inc., Bayer AG, Bristol‐Myers‐Squibb Co, and AstraZeneca Canada Inc.; and grant funding from AstraZeneca Canada Inc., Boehringer‐Ingelheim Canada Ltd., Pfizer Inc., and EMD Serono Inc. The remaining authors declare no conflicts of interest.

## ETHICAL APPROVAL

All experimental protocols were approved by the Sunnybrook Health Sciences Centre Research Ethics Board (SUN‐3774) with waiver of the requirement to obtain informed patient consent. All experimental methods were carried out under relevant guidelines and regulations.

## PATIENT CONSENT

Informed consent for patient information to be published in this article was not obtained as all patient data were anonymized.

## Data Availability

All data generated or analyzed during this study are included in this published article.
